# Supplementary material for: Characterizing the Pathogenicity and Immunogenicity of Simian Retrovirus Subtype 8 (SRV-8) Using SRV-8-Infected Cynomolgus Monkeys
Source: Viruses. 2023 Jul 12;15(7):1538. doi: 10.3390/v15071538 (PMC10384433; doi:10.3390/v15071538)
Supplement: Supplementary file 1 [file viruses-15-01538-s001.zip › viruses-2464167-supplementary.pdf]

Supplementary data

Manuscript Title: Characterizing the Pathogenicity and Immunogenicity of Simian Retrovirus subtype 8 (SRV-8) Using SRV-8 Infected Monkeys

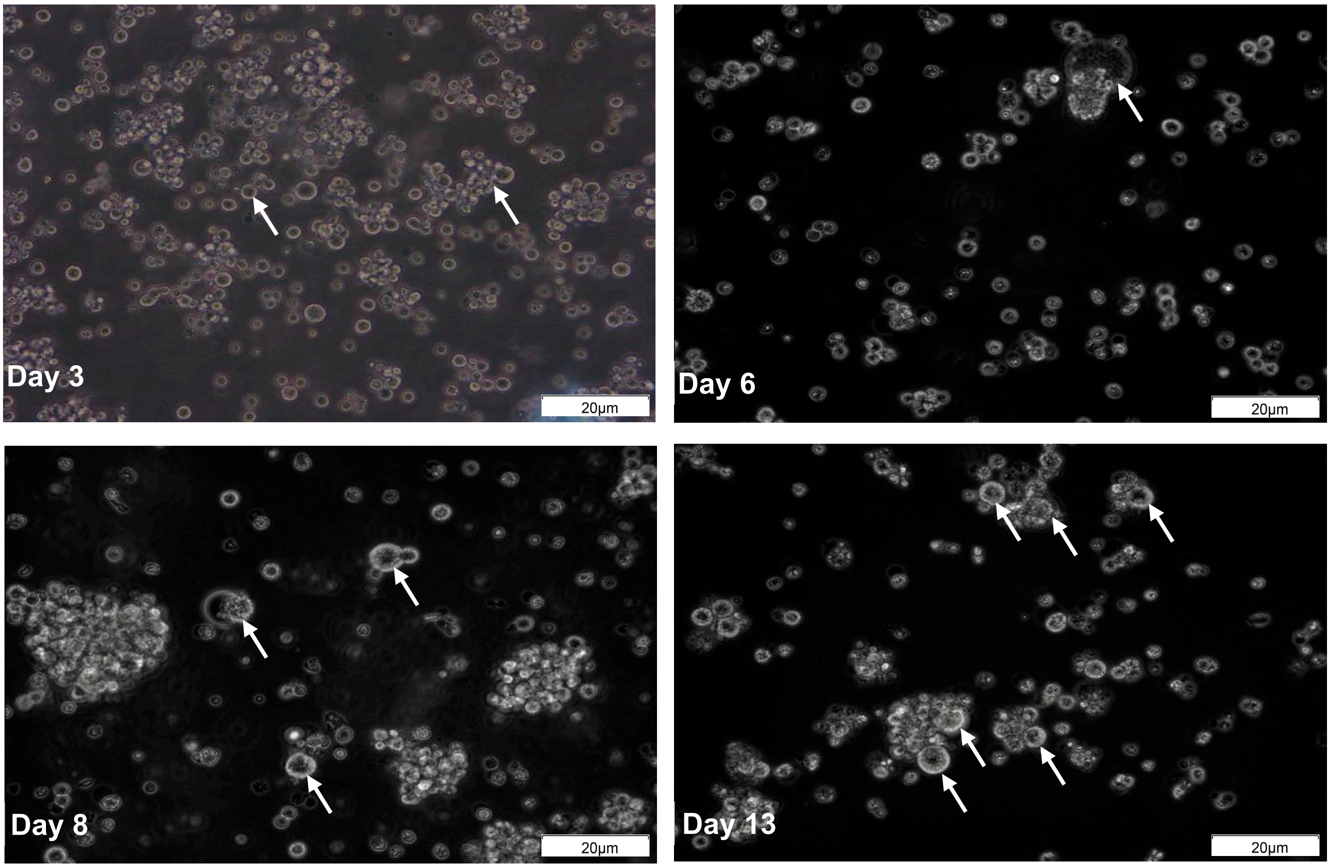

Figure. S1. Testing the cytopathic effect (CPE) of SRV-8 by incubating SRV-8-infected PBMCs with human B Raji cells.

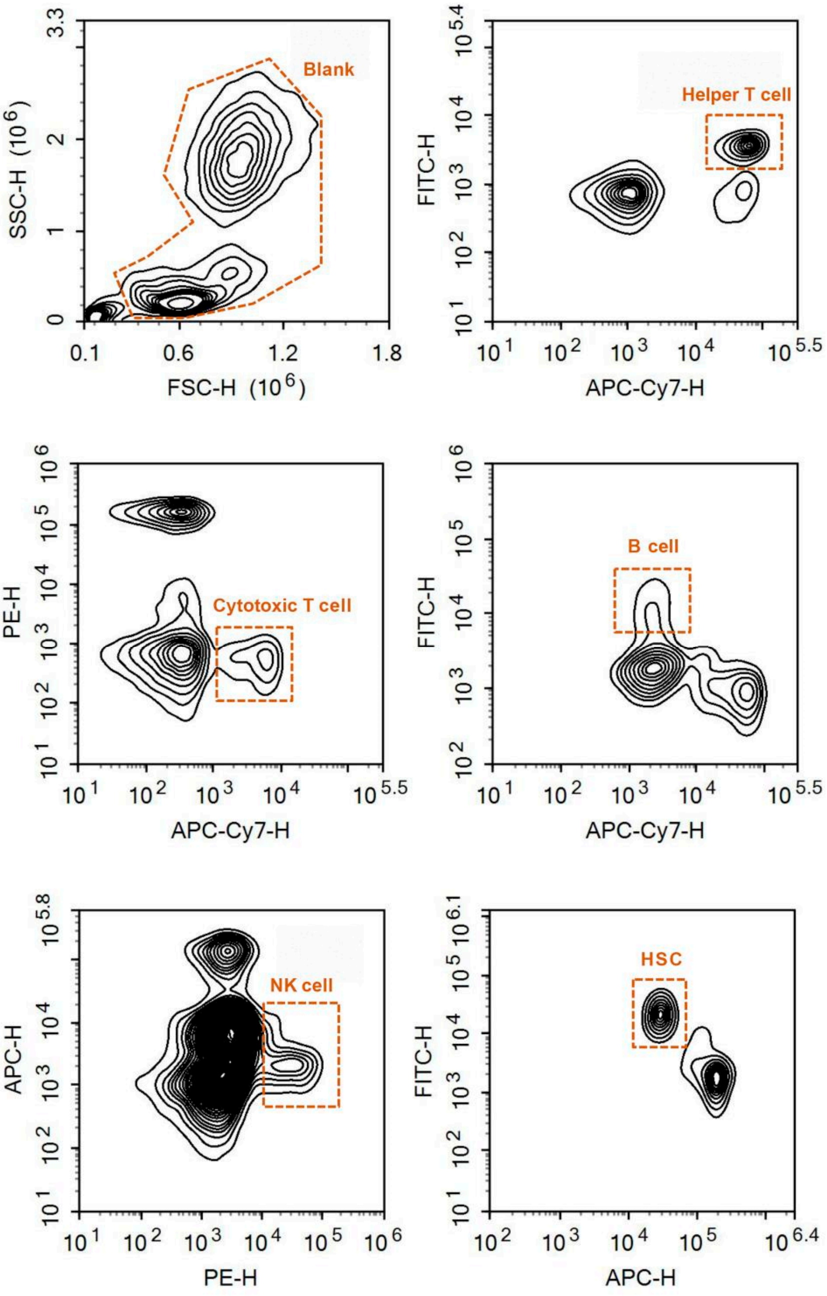

Figure S2: Flow cytometry immuno-phenotyping platforms are assembled to quantify the percentage of helper T cells, cytotoxic T cells, B cells, NK cells and HSC.

Table S1: The disease features of 120 female cynomolgus monkeys used in this work and the classification of these monkeys based on SRV-8 infection. NS, no significant changes; +, the monkey with the corresponding disease phenotypes or with the presence of provirus, virus particle, and antibody of SRV-8; -, the monkeys without the provirus, virus particle, and antibody of SRV-8.

Monkey number	Age (years)	Weight loss	Diarrhea	Anemia	SRV-8 Provirus (P)	Virus particle release (V)	Antibody production (A)	Group type[a]
1	9	NS	+	NS	-	-	-	/
2	9	+	NS	NS	-	-	-	/
3	10	NS	NS	+	-	-	-	/
4	10	NS	NS	+	-	-	-	/
5	10	NS	+	NS	-	-	-	/
6	8	+	+	NS	-	-	-	/
7	9	NS	+	NS	-	-	-	/
8	9	NS	NS	+	-	-	-	/
9	8	+	NS	NS	-	-	-	/
10	9	+	NS	NS	-	-	-	/
11	9	NS	+	NS	-	-	-	/
12	9	+	NS	NS	-	-	-	/
13	8	+	NS	NS	-	-	-	/
14	8	NS	+	NS	-	-	-	/
15	8	NS	+	NS	-	-	-	/
16	9	NS	+	NS	-	-	-	/
17	8	+	+	NS	+	-	-	P+V-A-
18	8	+	+	+	+	-	-	P+V-A-

19	9	+	+	+	+	-	-	P+V-A-
20	8	NS	+	+	+	-	-	P+V-A-
21	8	NS	NS	+	+	-	-	P+V-A-
22	8	+	+	+	+	-	-	P+V-A-
23	8	NS	+	+	+	-	-	P+V-A-
24	9	+	NS	+	+	-	-	P+V-A-
25	8	+	+	NS	+	-	-	P+V-A-
26	8	+	+	NS	+	-	-	P+V-A-
27	9	+	+	+	+	-	-	P+V-A-
28	8	+	+	+	+	-	-	P+V-A-
29	9	+	+	+	+	-	-	P+V-A-
30	8	+	+	+	+	+	-	P+V+A-
31	10	+	+	+	+	+	-	P+V+A-
32	10	+	+	+	+	+	-	P+V+A-
33	10	+	+	+	+	+	-	P+V+A-
34	9	+	+	+	+	+	-	P+V+A-
35	8	+	+	+	+	+	-	P+V+A-
36	8	+	+	+	+	+	-	P+V+A-
37	9	+	+	+	+	+	-	P+V+A-
38	9	+	+	+	+	+	-	P+V+A-
39	8	+	+	+	+	+	-	P+V+A-
40	9	+	+	+	+	+	-	P+V+A-
41	8	+	+	+	+	+	-	P+V+A-
42	8	+	+	+	+	+	-	P+V+A-
43	9	+	+	+	+	+	-	P+V+A-
44	9	+	+	+	+	+	-	P+V+A-

**45**	**9**	+	+	+	+	+	**-**	**P+V+A-**
**46**	**8**	**NS**	+	+	+	+	**-**	**P+V+A-**
**47**	**9**	+	**NS**	**NS**	+	+	**-**	**P+V+A-**
**48**	**9**	+	+	+	+	+	**-**	**P+V+A-**
**49**	**9**	+	+	**NS**	+	+	**-**	**P+V+A-**
**50**	**9**	+	+	+	+	+	**-**	**P+V+A-**
**51**	**8**	+	+	+	+	+	**-**	**P+V+A-**
**52**	**8**	**NS**	+	+	+	+	**-**	**P+V+A-**
**53**	**8**	**NS**	+	+	+	+	**-**	**P+V+A-**
**54**	**10**	+	+	+	+	+	**-**	**P+V+A-**
**55**	**10**	+	+	+	+	+	**-**	**P+V+A-**
**56**	**9**	+	+	+	+	+	**-**	**P+V+A-**
**57**	**8**	+	+	+	+	+	**-**	**P+V+A-**
**58**	**10**	+	**NS**	+	+	+	**-**	**P+V+A-**
**59**	**10**	+	+	+	+	+	**-**	**P+V+A-**
**60**	**10**	**NS**	+	+	+	+	**-**	**P+V+A-**
**61**	**9**	+	+	+	+	+	+	**P+V+A+**
**62**	**10**	+	+	+	+	+	+	**P+V+A+**
**63**	**9**	+	+	+	+	+	+	**P+V+A+**
**64**	**10**	+	+	+	+	+	+	**P+V+A+**
**65**	**8**	+	+	+	+	+	+	**P+V+A+**
**66**	**9**	+	+	+	+	+	+	**P+V+A+**
**67**	**9**	+	+	+	+	+	+	**P+V+A+**
**68**	**8**	+	+	+	+	+	+	**P+V+A+**
**69**	**9**	+	+	+	+	+	+	**P+V+A+**
**70**	**10**	**NS**	+	+	+	+	+	**P+V+A+**

71	9	+	+	NS	+	-	+	P+V-A+
72	8	+	+	NS	+	-	+	P+V-A+
73	10	NS	+	+	+	-	+	P+V-A+
74	10	NS	NS	+	+	-	+	P+V-A+
75	8	+	+	NS	+	-	+	P+V-A+
76	8	+	+	+	+	-	+	P+V-A+
77	9	+	+	+	+	-	+	P+V-A+
78	9	+	+	NS	+	-	+	P+V-A+
79	9	+	+	NS	+	-	+	P+V-A+
80	9	+	+	NS	+	-	+	P+V-A+
81	9	+	+	NS	+	-	+	P+V-A+
82	9	+	+	NS	+	-	+	P+V-A+
83	9	+	NS	NS	+	-	+	P+V-A+
84	8	+	NS	+	+	-	+	P+V-A+
85	9	+	NS	+	+	-	+	P+V-A+
86	10	+	+	+	+	-	+	P+V-A+
87	10	+	NS	+	+	-	+	P+V-A+
88	9	+	NS	+	+	-	+	P+V-A+
89	10	NS	+	+	+	-	+	P+V-A+
90	10	NS	+	+	+	-	+	P+V-A+
91	10	+	+	+	+	-	+	P+V-A+
92	9	NS	+	+	+	-	+	P+V-A+
93	8	+	+	+	+	-	+	P+V-A+
94	8	NS	+	+	+	-	+	P+V-A+
95	8	+	+	+	+	-	+	P+V-A+
96	10	+	+	NS	+	-	+	P+V-A+

**97**	**10**	+	+	+	+	-	+	**P+V-A+**
**98**	**8**	+	+	+	+	-	+	**P+V-A+**
**99**	**9**	+	+	**NS**	+	-	+	**P+V-A+**
**100**	**9**	**NS**	+	**NS**	-	-	+	**P-V-A+**
**101**	**9**	**NS**	**NS**	+	-	-	+	**P-V-A+**
**102**	**8**	**NS**	**NS**	+	-	-	+	**P-V-A+**
**103**	**10**	**NS**	+	+	-	-	+	**P-V-A+**
**104**	**8**	**NS**	**NS**	+	-	-	+	**P-V-A+**
**105**	**9**	**NS**	**NS**	+	-	-	+	**P-V-A+**
**106**	**9**	+	+	+	-	-	+	**P-V-A+**
**107**	**8**	+	**NS**	**NS**	-	-	+	**P-V-A+**
**108**	**8**	+	**NS**	**NS**	-	-	+	**P-V-A+**
**109**	**8**	**NS**	**NS**	+	-	-	+	**P-V-A+**
**110**	**10**	+	**NS**	+	-	-	+	**P-V-A+**
**111**	**10**	**NS**	+	+	-	-	+	**P-V-A+**
**112**	**8**	+	**NS**	+	-	-	+	**P-V-A+**
**113**	**10**	**NS**	**NS**	+	-	-	+	**P-V-A+**
**114**	**9**	**NS**	+	+	-	-	+	**P-V-A+**
**115**	**10**	**NS**	**NS**	+	-	-	+	**P-V-A+**
**116**	**8**	**NS**	**NS**	+	-	-	+	**P-V-A+**
**117**	**9**	**NS**	+	**NS**	-	-	+	**P-V-A+**
**118**	**8**	**NS**	+	+	-	-	+	**P-V-A+**
**119**	**8**	**NS**	+	**NS**	-	-	+	**P-V-A+**
**120**	**8**	+	+	+	+	+	-	**P+V+A-**

[a]P, provirus; V, released virus; A, antibody; the red words indicate the monkeys used in this work.

Table S2: The up- and down-regulated (fold change>4) genes in the monkey group P+V-A-, when compared with the group P-V-A-.

Gene name	BaseMean in P-V-A-	BaseMean in P+V-A-	Fold change (P+V-A-/P-V-A-)	P value
**Up-regulated in P+V-A-**				
ALAS2	44.1372	395.6063	8.9631	0.0000
MAP2	1.4255	22.0320	15.4552	0.0000
HUNK	13.8677	89.9257	6.4846	0.0001
ZNF462	19.9196	89.4587	4.4910	0.0001
TTLL7	7.2586	37.6248	5.1835	0.0002
C3	22.4336	91.3401	4.0716	0.0003
DEPDC1B	7.0825	34.9990	4.9416	0.0003
LOC101926697	2411.8218	24301.5770	10.0760	0.0004
LMOD2	11.3585	59.9700	5.2797	0.0006
CA1	27.4632	115.8360	4.2179	0.0008
SNCA	9.2909	69.5689	7.4878	0.0010
ATCAY	2.7725	19.2606	6.9469	0.0021
CCDC74B	5.7640	25.4719	4.4191	0.0022
BTNL3	17.1740	79.9365	4.6545	0.0037
CLEC4D	46.9425	320.4416	6.8263	0.0041
LOC102119091	103.8795	927.6430	8.9300	0.0042
LOC102135934	115.9682	596.0314	5.1396	0.0042
JHY	3.7572	19.0845	5.0794	0.0046
MAPK10	3.5268	18.9413	5.3707	0.0049
LOC102121016	0.5378	13.1191	24.3931	0.0050
LOC102120395	246.5004	1092.3742	4.4315	0.0063
HDC	11.9514	62.6409	5.2413	0.0067
LOC102141014	5.0668	42.9374	8.4743	0.0068
NR4A3	9.3726	68.9624	7.3579	0.0069
BPIFC	23.0564	106.3289	4.6117	0.0074
THBS1	1482.8237	6365.8214	4.2930	0.0076
PAQR9	2.6390	18.0155	6.8267	0.0079
WDR88	1.3643	11.6140	8.5128	0.0081
LOC123572755	3.4860	25.7372	7.3830	0.0087
MYO5C	16.7891	150.9380	8.9903	0.0090
SERPING1	2.9082	15.9306	5.4777	0.0096
SMO	7.8711	41.2830	5.2449	0.0102
TMEM212	0.4041	7.2665	17.9820	0.0118
LOC102144130	3.6577	16.8245	4.5998	0.0120
LOC102141414	5.3829	34.1376	6.3419	0.0122
LOC123572777	2.9331	14.9319	5.0908	0.0127
TBX20	0.8696	9.4379	10.8532	0.0133

LOC102134652	0.7748	8.5609	11.0487	0.0135
LOC102116245	91.7877	458.1123	4.9910	0.0140
SLC15A1	2.1487	12.5964	5.8623	0.0151
EPHA4	4.6354	18.8199	4.0600	0.0159
SMIM6	2.2935	12.6151	5.5003	0.0172
NFASC	4.7521	28.2974	5.9547	0.0173
LOC102142071	5.3208	38.6324	7.2607	0.0201
NOS2	0.3707	11.1005	29.9423	0.0203
CELF5	3.9053	15.9472	4.0835	0.0212
LOC102119621	2.2721	11.8224	5.2033	0.0239
ADM2	0.8042	7.6839	9.5543	0.0240
SLC13A3	9.0958	49.4544	5.4371	0.0240
DMRTB1	0.2243	5.4518	24.3034	0.0248
KLF1	1.9865	11.3512	5.7141	0.0261
CSRP2	2.6730	12.5470	4.6940	0.0264
PRSS12	3.4031	20.7259	6.0903	0.0296
CHRM4	0.5275	14.1605	26.8456	0.0303
CYP4F12	3.5560	17.9660	5.0524	0.0311
LOC102118436	1.9799	9.8741	4.9871	0.0327
LOC123572880	0.6730	7.9772	11.8539	0.0361
SLC44A4	0.4702	6.4858	13.7923	0.0378
RIMBP2	2.0189	10.2892	5.0965	0.0392
LOC107127060	0.2100	4.7928	22.8218	0.0415
SVEP1	1.3605	10.5330	7.7419	0.0429
ZDHHC19	0.1854	4.7108	25.4134	0.0433
CHAC1	1.1376	7.9303	6.9709	0.0448
RASEF	0.2100	4.4550	21.2132	0.0467
LOC123566747	1.1160	7.1002	6.3620	0.0473
LMOD1	3.0772	12.3198	4.0036	0.0474
LOC102136846	25.1554	327.3421	13.0128	0.0476
LOC102118535	0.4200	5.1186	12.1864	0.0490
LRRTM2	0.4486	5.4822	12.2196	0.0491
LOC123566813	30.1719	121.8870	4.0397	0.0495
**Down-regulated in P+V-A-**				
LOC123574098	73.5823	7.5010	0.1019	0.0000
CHSY3	37.8348	2.4316	0.0643	0.0000
LOC107130196	1430.6234	270.8976	0.1894	0.0000
CLDN7	109.4749	25.0117	0.2285	0.0000
CCDC153	477.3219	65.0599	0.1363	0.0000
MPZL2	201.5786	26.8552	0.1332	0.0001
FBP1	1098.1983	130.1615	0.1185	0.0001
ABCG4	25.3414	1.9837	0.0783	0.0002
ABCB11	88.9617	16.7115	0.1879	0.0002
MISP3	91.5628	20.6953	0.2260	0.0003

TBX18	26.0878	0.3025	0.0116	0.0004
RXRG	66.1266	12.6503	0.1913	0.0004
LOC102136128	7197.2328	1036.4262	0.1440	0.0005
LOC123566798	32.0253	7.1682	0.2238	0.0012
HIF3A	22.6245	1.6390	0.0724	0.0014
CPAMD8	337.7515	3.4493	0.0102	0.0015
CELSR1	4809.8461	478.5842	0.0995	0.0017
TCF7L1	225.7630	26.2360	0.1162	0.0022
C7H15orf48	399.5172	99.1921	0.2483	0.0024
LOC102128653	208.7444	41.2616	0.1977	0.0029
CPNE5	186.0879	40.8352	0.2194	0.0034
CERS4	28.6271	6.1740	0.2157	0.0035
PANX2	16.5922	2.5419	0.1532	0.0037
MYBPH	11.2575	0.6894	0.0612	0.0041
LOC123570349	1791.0124	299.0000	0.1669	0.0043
PLAU	240.0945	36.4828	0.1520	0.0045
CDHR2	374.1493	91.5499	0.2447	0.0062
CLTCL1	156.0330	23.2351	0.1489	0.0062
CCL15	9.9027	0.3025	0.0305	0.0071
NECTIN1	1168.1517	239.9873	0.2054	0.0083
HEY1	8.3193	0.3025	0.0364	0.0091
BDKRB2	559.0624	16.0645	0.0287	0.0094
PDZD3	334.8354	73.8953	0.2207	0.0095
FILIP1L	8.1087	0.3447	0.0425	0.0096
PRRG1	19.0507	3.9439	0.2070	0.0101
TCEAL2	9.9486	1.0177	0.1023	0.0106
NR1I2	7.8781	0.3025	0.0384	0.0112
BDKRB1	27.3142	1.2943	0.0474	0.0126
GUCY2C	54.5115	10.2728	0.1885	0.0126
FBP2	29.1499	0.7410	0.0254	0.0150
S100A12	68.5995	13.7807	0.2009	0.0153
LRRC46	57.3248	13.0324	0.2273	0.0156
LRRC3	19.1455	4.7858	0.2500	0.0158
LOC102145186	107.0916	24.4189	0.2280	0.0173
LOC102141933	112.2821	20.0531	0.1786	0.0178
TENM3	12.0343	1.0599	0.0881	0.0188
TUSC3	20.0509	4.3051	0.2147	0.0205
GPC4	72.8720	12.2259	0.1678	0.0210
LOC123566740	24.1941	0.6049	0.0250	0.0224
NTN3	9.4249	1.2779	0.1356	0.0261
CHI3L1	722.6851	93.5185	0.1294	0.0271
CDA	339.3152	70.1365	0.2067	0.0282
KCNK10	7.0879	0.3705	0.0523	0.0300
COL21A1	7.3600	0.3447	0.0468	0.0319

PBX1	71.2582	17.1408	0.2405	0.0322
DCN	10.4889	1.9837	0.1891	0.0324
LOC102123228	127.5268	1.2943	0.0101	0.0334
LTBP2	91.7259	19.6921	0.2147	0.0355
NAV3	84.7585	13.3421	0.1574	0.0356
BMP2	8.3884	0.9918	0.1182	0.0367
LOC123574606	10.6156	1.9415	0.1829	0.0377
SOWAHC	5.9742	0.3447	0.0577	0.0384
TRIM55	25.0379	4.1199	0.1645	0.0386
PGM5	14.2969	3.5640	0.2493	0.0397
SCRT2	11.5062	2.1172	0.1840	0.0420
C14H11orf52	11.2709	2.3120	0.2051	0.0428
SMIM38	13.7375	3.3132	0.2412	0.0441
WNT7B	21.7692	4.6920	0.2155	0.0453
MEGF10	6.8488	0.6894	0.1007	0.0459
AJAP1	61.2948	5.6277	0.0918	0.0485
CACNA1B	233.0397	58.0419	0.2491	0.0497

Table S3: Information of up-regulated gene and down-regulated gene in comparisons with P+V+A- and P-V-A- groups.

Gene name	BaseMean in P-V-A-	BaseMean in P+V+A-	Fold change (P+V+A-/P-V-A-)	P value
**Up-regulated in P+V+A-**				
FBXO39	3.9035	39.5257	10.1256	0.0000
IFI27	110.5829	10976.5090	99.2604	0.0000
CMPK2	81.6127	1159.7038	14.2098	0.0000
BCL2L14	16.2920	222.1350	13.6346	0.0000
ISG15	114.0817	3147.4964	27.5898	0.0000
RSAD2	161.7452	3678.5642	22.7430	0.0000
TNFAIP6	103.4224	477.7699	4.6196	0.0000
IFI44L	363.3144	4336.2589	11.9353	0.0000
APOBEC3A	213.1692	2864.5417	13.4379	0.0000
FOSL1	9.4822	51.0112	5.3797	0.0000
SERPING1	2.8439	121.6504	42.7763	0.0000
DDX60	482.6631	3231.8106	6.6958	0.0000
XAF1	239.4269	1007.3243	4.2072	0.0000
MOCOS	56.3175	244.4958	4.3414	0.0000
OAS2	1577.7149	12852.0878	8.1460	0.0000
KLHDC7B	129.5967	2022.7290	15.6079	0.0001
APOL2	3986.2114	16713.5152	4.1928	0.0001
GPC1	36.8315	429.6958	11.6665	0.0002
HSD3B7	15.3330	107.5797	7.0162	0.0002
C2	49.5893	495.6691	9.9955	0.0002
IFI44	1000.4738	6119.8476	6.1169	0.0002
IFI6	3492.8092	19861.6684	5.6864	0.0002
HERC5	707.5782	4742.6997	6.7027	0.0002
DEPDC1B	6.9187	28.4593	4.1134	0.0003
CXCL10	65.1426	753.0591	11.5602	0.0003
MX1	2516.7710	15090.4511	5.9960	0.0003
SHROOM4	80.2553	692.3446	8.6268	0.0004
APOL6	623.3065	4154.0495	6.6645	0.0004
IFIT1	1886.7034	13138.8605	6.9639	0.0004
LAG3	90.0483	403.3650	4.4794	0.0005
MSR1	10.8307	79.5685	7.3466	0.0005
TMPRSS2	139.2068	978.5182	7.0292	0.0006
HES4	107.1790	520.0650	4.8523	0.0008
MX2	2481.0937	11478.7339	4.6265	0.0008
SPATS2L	235.2622	1778.5168	7.5597	0.0008
ANTXRL	4.1843	20.1541	4.8166	0.0009
USP18	84.7955	917.7891	10.8236	0.0010

TLR3	24.9348	203.4546	8.1595	0.0010
HERC6	621.2770	3226.3320	5.1931	0.0011
MEIS3	17.2752	73.0220	4.2270	0.0011
IFIT3	3963.4605	19308.6742	4.8717	0.0021
BATF2	22.8209	157.7190	6.9112	0.0025
OAS1	116.0279	3649.5410	31.4540	0.0028
AK4	1.8493	11.6032	6.2743	0.0032
AGRN	203.0202	936.0474	4.6106	0.0045
CCL8	0.3581	9.6107	26.8348	0.0046
ADM2	0.7864	8.1127	10.3157	0.0047
IFIT2	3033.2792	26469.6423	8.7264	0.0052
APOBEC3B	20.5056	173.1189	8.4425	0.0055
DZIP1L	8.5613	151.2792	17.6700	0.0060
APOL4	48.1440	283.1222	5.8807	0.0060
CXCL9	6.3546	28.2888	4.4517	0.0063
RSPH1	1.1867	8.7612	7.3827	0.0067
SIGLEC1	266.2647	3314.1973	12.4470	0.0071
LOC102135697	4.3553	25.3196	5.8135	0.0075
TWIST1	2.3471	16.8718	7.1882	0.0078
LOC123568345	0.6335	7.3743	11.6408	0.0084
ABCC9	0.7824	7.2585	9.2776	0.0097
HPGD	3.5829	43.2684	12.0762	0.0100
LOC102122172	2.8043	13.8062	4.9232	0.0104
DLGAP3	1.3399	9.0802	6.7766	0.0104
CLEC4D	45.8727	356.3990	7.7693	0.0104
TRIL	3.7359	18.4441	4.9370	0.0108
OLFML2B	4.5265	32.0059	7.0708	0.0113
HYDIN	2.3012	10.6044	4.6082	0.0113
CKB	2.5377	11.7271	4.6212	0.0114
CXCL11	0.6095	16.6905	27.3819	0.0116
TFPI2	1.5039	12.4521	8.2797	0.0118
IL21	7.2407	30.5190	4.2149	0.0119
LOC123568677	0.3671	5.6119	15.2870	0.0124
CCL7	0.2141	4.8878	22.8242	0.0124
CXCL12	1.3769	14.3532	10.4241	0.0130
KCTD14	28.1453	185.4943	6.5906	0.0135
SCUBE2	2.4176	11.2054	4.6350	0.0159
PTK7	34.7327	170.1738	4.8995	0.0163
BAIAP2L1	1.4759	8.2883	5.6157	0.0167
DHRS2	0.3059	6.4385	21.0474	0.0175
LOC102142596	257.1516	1307.9134	5.0862	0.0183
ACKR1	11.2297	52.6139	4.6852	0.0200
LOC102136192	77.5936	786.8649	10.1408	0.0202
RIN2	21.2311	86.7600	4.0865	0.0209

LOC102119621	2.2187	10.1673	4.5826	0.0212
LRP6	0.3720	5.1738	13.9088	0.0213
CFB	2.8658	13.1194	4.5779	0.0218
CCL22	13.0411	74.4771	5.7110	0.0228
ITGA8	2.0891	16.0058	7.6615	0.0229
HDC	11.6844	64.5307	5.5228	0.0247
HUNK	13.5539	70.2730	5.1847	0.0254
LOC102142071	5.1961	21.5963	4.1563	0.0255
ADM	22.6980	147.7873	6.5110	0.0258
DMRT2	1.1394	10.3183	9.0562	0.0272
MYO7B	53.1318	248.3411	4.6741	0.0276
TTC39A	44.2707	197.7545	4.4669	0.0281
SYCE3	0.1812	4.2624	23.5174	0.0303
NEURL3	1.7024	8.2137	4.8247	0.0306
LOC101926697	2355.9674	25741.6410	10.9261	0.0310
FCGR1A	142.3124	589.3016	4.1409	0.0313
LOC102119091	101.6303	415.3245	4.0866	0.0320
AMPH	0.1812	3.8662	21.3313	0.0330
TMOD1	1.6083	10.1781	6.3284	0.0331
PKD1L1	1.2103	13.4823	11.1394	0.0345
ANTXR1	0.6335	7.9952	12.6208	0.0361
LCN2	85.9293	558.4570	6.4990	0.0362
CDKN1C	446.3575	1917.2098	4.2952	0.0368
TBX20	0.8496	9.4555	11.1290	0.0369
DNAAF3	3.4609	16.0586	4.6401	0.0377
CTSG	21.6225	109.7539	5.0759	0.0386
ALAS2	43.1103	622.4220	14.4379	0.0389
PLAAT2	2.9184	14.8398	5.0850	0.0396
MPO	73.2047	305.8130	4.1775	0.0408
SNCG	0.1530	5.4534	35.6542	0.0449
ELANE	24.2483	106.4406	4.3896	0.0469
ST8SIA5	0.5723	6.4887	11.3380	0.0471
HPD	1.4098	6.5880	4.6729	0.0478
**Down-regulated in P+V+A-**				
LOC123574720	125.0900	29.9234	0.2392	0.0000
LRRN1	547.7172	65.6867	0.1199	0.0000
SYNDIG1	47.3532	9.9207	0.2095	0.0000
PPP1R9A	46.8006	4.7120	0.1007	0.0000
LOC107130176	69.3449	14.4195	0.2079	0.0002
SHISA8	29.9348	7.0997	0.2372	0.0002
LOC102130471	31.3855	5.9207	0.1886	0.0006
EYA2	48.5426	4.9314	0.1016	0.0007
CDH2	13.8909	1.9334	0.1392	0.0012
GRIA1	18.5284	4.4134	0.2382	0.0015

DRD1	29.0689	3.5516	0.1222	0.0019
ZBTB8A	271.2463	48.6134	0.1792	0.0023
LOC102123570	50.8156	11.5963	0.2282	0.0031
CNR1	9.7290	1.1536	0.1186	0.0039
LOC102124366	14.8211	3.3500	0.2260	0.0042
DCHS2	7.5089	0.5784	0.0770	0.0048
LOC102141933	109.7118	27.3801	0.2496	0.0069
COBLL1	16.0928	3.5657	0.2216	0.0075
EFNA2	9.6547	1.4388	0.1490	0.0084
TBX18	25.4906	6.1306	0.2405	0.0092
MYBPHL	132.2445	28.4105	0.2148	0.0093
RBM20	11.6849	2.5784	0.2207	0.0096
TACSTD2	6.1446	0.4702	0.0765	0.0097
LRRC18	9.6684	1.8269	0.1890	0.0107
TENT5B	10.7245	0.9657	0.0900	0.0115
CACNA1B	227.7625	52.5233	0.2306	0.0118
LGR5	12.3268	2.9249	0.2373	0.0141
MYBPH	11.0027	2.1849	0.1986	0.0147
LOC123567067	7.3306	1.0159	0.1386	0.0149
XCR1	10.0264	1.9508	0.1946	0.0154
RFTN2	7.1257	0.1610	0.0226	0.0169
TEX9	16.5286	1.8689	0.1131	0.0171
INSM1	5.7419	0.4558	0.0794	0.0185
LOC102143181	11.4905	2.4075	0.2095	0.0192
CAP2	18.2147	1.6090	0.0883	0.0200
GCSAM	10.2527	2.1036	0.2052	0.0227
STAC2	12.8914	2.9098	0.2257	0.0230
LAMA5	3365.2346	400.1941	0.1189	0.0236
LOC102145452	6.0709	0.8775	0.1446	0.0237
PPFIA2	17.4502	3.0504	0.1748	0.0246
SCRT2	11.2434	2.7266	0.2425	0.0254
BDKRB2	546.0156	69.7031	0.1277	0.0262
CPAMD8	330.2739	54.1287	0.1639	0.0297
LOC123572190	31.0600	0.5552	0.0179	0.0298
SERPINH1	7.0407	1.1018	0.1565	0.0304
ADPRHL1	7.1252	1.2259	0.1721	0.0324
AKR7A3	4.0778	0.3328	0.0816	0.0353
LOXL1	11.7963	1.7660	0.1497	0.0358
ALDH1L1	7.0855	1.4365	0.2027	0.0372
SERINC2	6.0160	0.7401	0.1230	0.0405
PRRT1	31.0940	4.9446	0.1590	0.0414
IL12B	40.7570	4.9138	0.1206	0.0416
LOC102124761	6.4841	1.0877	0.1677	0.0421
FAM162B	8.4300	0.3229	0.0383	0.0497

Table S4: Information of up-regulated gene and down-regulated gene in comparisons with P+V+A+ and P-V-A- groups.

Gene name	BaseMean in P-V-A-	BaseMean in P+V+A+	Fold change (P+V+A+/P-V-A-)	P value
**Up-regulated in P+V+A+**				
LOC123575142	57.8738	482.4341	8.3360	0.0000
LOC102134910	39.7723	165.6358	4.1646	0.0000
NR4A2	424.8059	1932.2457	4.5485	0.0000
LOC102119091	106.9400	878.9759	8.2193	0.0000
PNMT	66.3296	693.8350	10.4604	0.0000
NR4A3	9.6750	57.3335	5.9259	0.0001
LOC107130742	5.6940	82.5255	14.4933	0.0002
KEG98_p07 (COX3)	6187.3223	27713.0036	4.4790	0.0002
LOC102122868	36.7951	152.1704	4.1356	0.0002
CNGA1	10.6322	61.0300	5.7401	0.0004
WDR88	1.4081	20.2156	14.3571	0.0006
MSR1	11.4196	53.0273	4.6435	0.0008
LOC102131482	9.5486	41.4039	4.3361	0.0010
LOC102134507	11.4221	58.2759	5.1020	0.0011
LOC102127471	7.5360	32.6076	4.3269	0.0049
LOC107129821	17.6008	71.7336	4.0756	0.0056
RGS1	520.2490	2191.3465	4.2121	0.0062
RGS13	4.1619	24.5823	5.9065	0.0063
SREK1IP1	36.4864	238.8534	6.5464	0.0083
LOC102115461	2.5661	16.3958	6.3895	0.0107
ADARB2	2.2242	15.6348	7.0294	0.0124
LOC102135811	3.7893	20.2358	5.3402	0.0138
AK1	7.6827	68.3093	8.8914	0.0150
EPOP	9.3339	44.5536	4.7733	0.0165
KEG98_p12 (ND2)	814.5374	5418.1691	6.6518	0.0174
LOC123567073	6.9841	32.7915	4.6952	0.0186
ADM2	0.8280	9.8395	11.8836	0.0197
CD69	530.8168	2520.8207	4.7489	0.0217
LPAR3	1.6113	11.8452	7.3513	0.0269
LOC102147128	39.8801	211.0483	5.2921	0.0284
ABO	20.1052	98.8003	4.9142	0.0304
ONECUT3	0.4621	26.5703	57.4974	0.0334
FBXO2	5.9686	29.4982	4.9423	0.0338
LOC102129332	4.9014	19.7193	4.0232	0.0352
C4H6orf163	1.6687	11.7041	7.0137	0.0356
C14H11orf16	0.7854	8.4814	10.7992	0.0383
LOC107130326	1.6652	10.8322	6.5050	0.0385

OSTN	4.9946	30.8664	6.1800	0.0421
LOC102135781	10.7921	52.4200	4.8573	0.0428
ASTL	1.8276	12.3416	6.7527	0.0468
KEG98_p02 (ND6)	3.9342	36.8961	9.3783	0.0489
**Down-regulated in P+V+A+**				
TIMP2	1234.0138	306.9943	0.2488	0.0000
ZBED6	477.2033	72.1949	0.1513	0.0000
ZNF852	52.0205	9.0181	0.1734	0.0001
LOC107127181	22.4828	1.4085	0.0626	0.0009
NIPSNAP3B	74.1911	18.3007	0.2467	0.0009
LOC123569555	210.1290	45.4052	0.2161	0.0012
IL4I1	64.5310	14.6320	0.2267	0.0012
PDK4	789.2240	129.7728	0.1644	0.0014
LOC107128162	33.3338	5.6845	0.1705	0.0014
LOC102141933	115.6499	4.2155	0.0365	0.0015
ENTPD2	39.7974	1.4287	0.0359	0.0019
MPZL2	207.6939	34.9887	0.1685	0.0022
LOC102143631	2995.4188	588.4838	0.1965	0.0023
TCL1A	119.4579	16.5066	0.1382	0.0024
CHRD	49.8881	8.4511	0.1694	0.0032
CUX2	39.0769	8.9576	0.2292	0.0035
ZNF285	16.2973	0.9525	0.0584	0.0036
HEPACAM2	51.7345	9.8798	0.1910	0.0042
LOC102124408	386.4678	84.6901	0.2191	0.0045
HIF3A	23.3146	1.3984	0.0600	0.0051
PRIMA1	76.3838	7.6095	0.0996	0.0056
LOC102118713	16.1054	1.4186	0.0881	0.0057
TBX18	26.9063	0.9424	0.0350	0.0076
CPM	217.4349	51.5583	0.2371	0.0095
HAS1	335.1793	54.3931	0.1623	0.0136
FFAR4	29.6011	2.3307	0.0787	0.0150
ZNF311	21.1373	4.2558	0.2013	0.0153
HERC2	67.8476	13.6795	0.2016	0.0167
DRD1	30.6222	1.9049	0.0622	0.0173
CLEC9A	23.5321	5.6442	0.2398	0.0183
EPHA2	9.9904	0.4661	0.0467	0.0185
TMPPE	26.2950	5.2183	0.1985	0.0192
CPNE5	191.8330	44.9415	0.2343	0.0205
LOC107128858	9.6238	0.4661	0.0484	0.0212
SERPINF2	29.0838	6.0901	0.2094	0.0223
LOC102122644	21.4788	4.2356	0.1972	0.0231
KCNB1	15.0765	0.4762	0.0316	0.0254
GCSAM	10.8086	0.9323	0.0863	0.0265
CTTNBP2NL	29.9819	5.2082	0.1737	0.0269

APOA2	30.9083	7.1232	0.2305	0.0270
ENTPD3	1047.3531	221.2711	0.2113	0.0293
SMIM38	14.1576	1.9049	0.1345	0.0307
OSCP1	17.0956	3.2933	0.1926	0.0309
TRIM67	127.1026	26.4771	0.2083	0.0326
NRP1	122.8047	8.0051	0.0652	0.0344
TCL1B	22.3955	3.7796	0.1688	0.0371
PLAU	247.2603	53.3297	0.2157	0.0411
STBD1	14.0400	2.3711	0.1689	0.0411
FAT2	14.9543	0.9525	0.0637	0.0420
EHF	16.5765	1.8948	0.1143	0.0423
SFRP2	8.3262	0.4762	0.0572	0.0433
LOC102122391	36.0832	6.1405	0.1702	0.0437
MYBPHL	139.3567	17.8748	0.1283	0.0456
PROK2	452.5603	110.4793	0.2441	0.0472
LOC102143181	12.1301	0.9323	0.0769	0.0499

Table S5: Information of up-regulated gene and down-regulated gene in comparisons with P+V-A+ and P-V-A- groups.

Gene name	BaseMean in P-V-A-	BaseMean in P+V-A+	Fold change (P+V-A+/P-V-A-)	P value
**Up-regulated in P+V-A+**				
NR4A2	397.0469	2982.9925	7.5129	0.0000
NR4A3	9.0472	73.9384	8.1725	0.0000
LOC123572755	3.3616	27.4876	8.1770	0.0000
CLEC5A	14.6752	62.9655	4.2906	0.0000
SEMA3A	8.1230	38.8698	4.7852	0.0000
TNNT3	113.9255	502.2333	4.4084	0.0000
CGA	6.9774	33.3366	4.7778	0.0000
CCDC89	7.3295	51.8221	7.0704	0.0000
PRSS12	3.2775	20.7798	6.3402	0.0001
LOC102119091	99.9594	660.2617	6.6053	0.0001
TCTE1	3.9010	28.6392	7.3414	0.0001
CHRM4	0.5099	9.5576	18.7452	0.0004
MROH8	5.8523	23.5423	4.0227	0.0010
SLC1A2	3.4175	19.1076	5.5912	0.0010
SLC13A2	1.4129	12.6478	8.9515	0.0011
LOC102139829	0.3812	9.6219	25.2391	0.0012
LOC123571204	35.4862	169.0576	4.7640	0.0014
GPR176	3.4357	16.0246	4.6641	0.0026
SPAG6	3.1631	15.7616	4.9829	0.0029
LRRTM2	0.4295	7.1239	16.5874	0.0052
MEIS3	17.0035	117.0028	6.8811	0.0053
WDR88	1.3163	9.4297	7.1638	0.0055
LGI4	11.5211	51.0218	4.4286	0.0066
RASSF6	24.3733	115.1407	4.7240	0.0074
LOC102133967	1.3621	8.7045	6.3903	0.0087
TENM1	52.3695	255.8973	4.8864	0.0089
KCNQ4	2.0133	10.2328	5.0826	0.0124
LMOD3	0.9267	10.0292	10.8223	0.0135
LOC102141014	4.8773	30.6568	6.2856	0.0137
LOC107127636	2.6701	11.0066	4.1221	0.0161
REEP1	14.7848	66.5514	4.5014	0.0199
HYDIN	2.2612	9.2446	4.0883	0.0216
LOC123571731	0.7581	18.9845	25.0409	0.0217
CNBD2	1.2880	7.4373	5.7742	0.0224
MAGI1	19.1953	79.3189	4.1322	0.0262
CDNF	0.8956	6.4483	7.1999	0.0284
FXYD1	1.2381	7.1300	5.7589	0.0317

LOC102115526	7.9325	52.2822	6.5909	0.0339
LOC123569497	1.6552	7.8142	4.7211	0.0355
FAM181A	1.4789	7.4466	5.0351	0.0369
IQSEC3	8.2840	55.0861	6.6497	0.0373
STPG3	0.8718	6.1592	7.0646	0.0373
NEURL3	1.6788	7.6354	4.5481	0.0398
PDZRN3	110.5462	459.3932	4.1557	0.0399
LOC107127641	0.1791	3.6217	20.2194	0.0412
PPP4R4	2.3465	9.6801	4.1254	0.0413
PIGR	1.5734	8.1552	5.1834	0.0464
TNFSF11	1.1243	6.1627	5.4813	0.0479
**Down-regulated in P+V-A+**				
LOC102118223	192.5301	21.9601	0.1141	0.0000
PDK4	737.1465	159.3183	0.2161	0.0000
HSPA1A	926.9256	224.1926	0.2419	0.0001
TBX18	25.1172	2.1907	0.0872	0.0006
DRD1	28.6033	2.3108	0.0808	0.0007
PTX3	371.3682	76.3965	0.2057	0.0008
UPK1A	14.1441	2.3786	0.1682	0.0009
SCRT2	11.0712	1.0776	0.0973	0.0012
LOC102141933	107.9609	18.7587	0.1738	0.0012
FOSB	1873.3219	428.3141	0.2286	0.0013
CDHR5	24.6088	5.3867	0.2189	0.0015
DNASE2B	20.0715	3.3857	0.1687	0.0021
TMEM178B	15.3048	2.0876	0.1364	0.0025
CLTCL1	150.0874	33.5280	0.2234	0.0026
TCF7L1	217.1840	42.9796	0.1979	0.0029
KCTD15	15.8264	2.9599	0.1870	0.0030
CHST13	28.8017	5.3309	0.1851	0.0059
DEFB1	5.1462	0.1991	0.0387	0.0060
COL21A1	7.0765	0.4847	0.0685	0.0069
NKX6-1	6.4443	0.4911	0.0762	0.0084
EPHB3	25.6413	1.6450	0.0642	0.0098
LOC107128858	8.9922	1.5716	0.1748	0.0115
IL1B	1255.4017	278.6908	0.2220	0.0146
TACSTD2	6.0662	0.6429	0.1060	0.0151
LOC102123544	7.0493	0.5630	0.0799	0.0185
CNGB3	4.2091	0.1702	0.0404	0.0191
CPNE4	23.5240	0.7161	0.0304	0.0197
LOC107130823	95.2437	5.1799	0.0544	0.0209
BDKRB2	537.3915	73.0219	0.1359	0.0224
LOC123566740	23.2942	3.1059	0.1333	0.0287
KCNB1	14.0595	2.9215	0.2078	0.0290
LOC107128043	6.3073	1.0552	0.1673	0.0294

LAMC2	6.6482	1.1748	0.1767	0.0342
LOC102131482	8.9049	2.0869	0.2344	0.0346
BDKRB1	26.2805	5.3515	0.2036	0.0352
IL12B	40.0338	4.4525	0.1112	0.0367
AJAP1	59.1453	9.9384	0.1680	0.0389
TNFSF15	15.6352	3.2438	0.2075	0.0428
KCNK1	6.3626	1.3733	0.2158	0.0446
LYPD8	140.8722	29.3667	0.2085	0.0498

Table S6: Information of up-regulated gene and down-regulated gene in comparisons with P-V-A+ and P-V-A- groups.

Gene name	BaseMean in P-V-A-	BaseMean in P-V-A+	Fold change (P-V-A+/P-V-A-)	P value
**Up-regulated in P-V-A+**				
LOC102119091	99.0927	645.9367	6.5185	0.0000
CA1	26.1586	123.9242	4.7374	0.0000
MSR1	10.5269	47.3325	4.4963	0.0000
CTH	1.4155	15.1307	10.6896	0.0001
LOC102140314	2.5592	16.6355	6.5001	0.0002
SNCG	0.1499	5.8185	38.8208	0.0028
MAST1	3.7676	17.8632	4.7413	0.0029
PRSS12	3.2494	14.2262	4.3781	0.0039
FOSL1	9.2202	44.1792	4.7916	0.0044
CLEC5A	14.5552	68.8438	4.7299	0.0051
CLEC4D	44.6811	179.4003	4.0151	0.0061
LOC102133967	1.3504	8.6762	6.4251	0.0069
ALPL	622.6676	4425.6725	7.1076	0.0072
LOC102122172	2.7422	14.8614	5.4196	0.0076
ADM2	0.7683	6.6586	8.6661	0.0092
DMRTB1	0.2110	4.8014	22.7549	0.0126
LOC123572755	3.3268	18.0007	5.4109	0.0132
LOC123572777	2.7916	11.3252	4.0568	0.0139
HUNK	13.1968	63.6147	4.8205	0.0139
OTOF	1.5653	7.9605	5.0855	0.0158
GRM2	2.3736	10.5188	4.4315	0.0171
DMRT2	1.1078	7.6669	6.9205	0.0184
NR4A3	8.9557	55.0761	6.1499	0.0193
UPK1B	5.7502	26.4274	4.5959	0.0219
LOC123566792	0.8320	6.3183	7.5942	0.0230
AK4	1.7999	7.9071	4.3931	0.0243
LOC102130668	0.2011	6.7652	33.6355	0.0245
ST8SIA5	0.5597	5.5246	9.8710	0.0251
ALAS2	42.1030	232.6421	5.5255	0.0254
ABCB1	20.2490	111.0010	5.4818	0.0263
SCN5A	0.7058	11.2926	15.9991	0.0277
LOC123571204	35.1110	156.3726	4.4537	0.0298
IL12A	1.0191	5.8058	5.6970	0.0381
LIFR	0.7371	5.1454	6.9807	0.0419
LOC102121053	2.8785	11.6266	4.0392	0.0431
CHRM4	0.5047	7.4538	14.7689	0.0436
LOC102139826	0.4496	6.2231	13.8401	0.0444

CXCL11	0.5947	4.3642	7.3380	0.0459
ND4L	245.8093	1238.2840	5.0376	0.0476
**Down-regulated in P-V-A+**				
TCL1A	110.3442	20.1371	0.1825	0.0000
MYBPH	10.7047	1.4174	0.1324	0.0036
LOC102141490	15.1294	3.2282	0.2134	0.0037
CACNA1B	222.7122	37.7028	0.1693	0.0039
TCL1B	20.7185	4.3573	0.2103	0.0040
GPT2	261.5631	56.0300	0.2142	0.0072
EHF	15.3667	2.6393	0.1718	0.0092
LOC102143181	11.2048	1.8441	0.1646	0.0092
LOC123573583	8.6695	1.5173	0.1750	0.0126
LOC102123544	6.9758	0.3182	0.0456	0.0128
NXNL2	14.6346	2.4035	0.1642	0.0130
PLXDC1	10.3889	2.5115	0.2417	0.0169
TNR	4.2453	0.1575	0.0371	0.0174
LOC101867510	16.3562	1.1714	0.0716	0.0181
BFSP2	7.3783	1.1478	0.1556	0.0198
CYP11A1	12.0650	0.1575	0.0131	0.0251
NPTXR	5.1693	0.7124	0.1378	0.0333
TENT5B	10.4356	2.3197	0.2223	0.0405
LOC102118330	4.5058	0.5290	0.1174	0.0417
HOXC5	3.2792	0.1501	0.0458	0.0459
